# Conference Calendar

**DOI:** 10.1002/cfg.480

**Published:** 2005-06


					Comparative and Functional Genomics
Comp Funct Genom 2005; 6: 256-258.
Published online in Wiley InterScience (www.interscience.wiley.com). DOI: 10.1002/cfg.480
Conference Calendar
October-December 2005
Animal Models
CSHL/WT -- Functional Genomics of
Mammalian Nervous Systems, September
28-October 2
http://meetings.cshl.edu/meetings/neugenuk05.
shtml
CHI -- Disease Models, October 18-19
http://www.discoveryontarget.com/05anm.asp
IMGC-19th Annual Meeting, November 5-8
http://www.imgs.org/cal.shtml
CSHL -- Rat Genomics and Models,
December 8-11
http://meetings.cshl.edu/meetings/rodent05.
shtml
Bioinformatics
European Conference on Computational
Biology (ECCB), September 28-October 1
http://www.eccb05.org/
5th Workshop on Algorithms in
Bioinformatics, October 3-6
http://www.lsi.upc.edu/wabi05
Workshop on Statistics in Genomics and
Proteomics, October 5-8
http://wsgp.deio.fc.ul.pt/
Information Technologies in Agriculture, Food
and Environment, October 12-14
http://itafe05.cukurova.edu.tr/Default.aspx
5th Virtual Conference on Genomics &
Bioinformatics, October 25-28
http://www.virtualgenomics.org/vcgb/confe-
rence 2005.htm
CSHL/WT -- Genome Informatics, October
26-30
http://meetings.cshl.edu/meetings/info05.shtml
Comparative and Functional Genomics (BITS)
Workshop, October 27-30
http://www.wellcome.ac.uk/doc WTX024 428.
html
TIGR/JAX -- 8th Annual Conference on
Computational Genomics, November 9-12
http://www.jax.org/courses/events/course-
details.do?id=137
IEEE -- Symposium on Computational
Intelligence in Bioinformatics and
Computational Biology, November 14-15
http://ieee-cis.org/cibcb2005/
Functional Genomics
CSHL -- RNAi, September 28-October 2
http://meetings.cshl.edu/meetings/rnai05.shtml
CHI -- RNAi, October 19-20
http://www.discoveryontarget.com/05rnid.asp
Human Genomics and Genetics
ASHG-55th Annual Meeting, October 25-29
http://genetics.faseb.org/genetics/ashg/menu-
annmeet.shtml
Copyright  2005 John Wiley & Sons, Ltd.
Conference Calendar 257
General
3rd International Congress on Developmental
Origins of Health & Disease, November 16-20
http://www.mpi-evv.com/2005DOHaD/frameset.
htm
FAOBMB -- Genomics and Proteomics in
Health and Agriculture, November 20-23
http://www.18faobmbsymp.com/
Metabolomics
CHI -- Metabolic Profiling, December 7-8
http://www.healthtech.com/conference-fall.
asp
CHI -- Analytical Methods for Metabolic
Profiling, December 9
http://www.healthtech.com/conference-fall.asp
Microbes
ASM -- Pasteurellaceae 2005, October 23-26
http://www.asm.org/Meetings/index.asp?bid=
27 649
ESF/EMBO -- Comparative Genomics of
Eukaryotic Microorganisms: Eukaryotic
Genome Evolution, Approaches with Yeasts
and Fungi, November 12-17
http://www.esf.org/conferences/lc05196
Pharmacogenomics/Genomics and
Disease
WT -- Neurogenomics, September
28-October 2
http://www.wellcome.ac.uk/doc wtd003175.html
CHI -- Discovery on Target, October 18-21
http://www.discoveryontarget.com/
IBC -- EuroTIDES 2005, December 6-8
http://www.ibc-lifesci.com/eurotides
Plants
Genomics in Business: the Agro-Food Industry,
October 2-4
http://www.genomicsinbusiness.nl/
ASPB -- Plant Genetics 2005, October 12-16
http://www.aspb.org/meetings/pg-2005/
7th International Wheat Conference,
November 27-December 2
http://www.7iwc.com.ar/index.php
Proteomics
HUPO-3rd Annual World Congress:
Proteomics: Decoding the genome, October
25-27
http://www.hupo2004.cn/
Systems Biology
6th International Conference on Systems
Biology, October 19-22
http://www.icsb-2005.org/
Transcriptomics/Regulation
CHI -- Microarray Data Validation, October
6-7
http://www.healthtech.com/2005/qpe/index.asp
IGB -- Epigenetic Bases of Genome
Reprogramming, October 8-11
http://w3.igb.cnr.it/workshop/Main.htm
CHI -- Gene Regulation and Regulatory
Networks, October 17
http://www.discoveryontarget.com/05rsq.asp
Copyright  2005 John Wiley & Sons, Ltd. Comp Funct Genom 2005; 6: 256-258.
258 Conference Calendar
Biotech Industry
6th European Biotechnology Symposium,
November 13-15
http://www.bioconferences.com/ebs/
Key:
ASHG: American Society of Human Genetics:
http://genetics.faseb.org/genetics/ashg/
ashgmenu.htm
ASPB: American Society of Plant Biologists:
http://www.aspb.org/
ASM: American Society for Microbiology:
http://www.asm.org/
CHI: Cambridge Healthtech Institute:
http://www.healthtech.com
CSHL: Cold Spring Harbor Laboratories:
http://meetings.cshl.org/index.htm
EMBO: European Molecular Biology Organiza-
tion: http://www.embo.org/
ESF: European Science Foundation:
http://www.esf.org/
FAOBMB: Federation of Asian and Oceanian Bio-
chemists and Molecular Biologists:
http://www.bic.nus.edu.sg/faobmb/home.html
HUPO: Human Proteome Organisation:
http://www.hupo.org
IBC: IBC Conferences: http://www.ibc-lifesci.com
IEEE: Institute of Electrical and Electronics Engi-
neers: http://www.ieee.org/
IGB: Institute of Genetics and Biophysics:
http://www.igb.cnr.it/
IMGC: International Mammalian Genome Society:
http://www.imgs.org/index.shtml
JAX: The Jackson Laboratory: http://www.jax.
org/
TIGR: The Institute for Genomic Research:
http://www.tigr.org
WT: Wellcome Trust: http://www.wellcome.
ac.uk/
The Conference Calendar is a listing of forthcoming conferences of interest to our readership. We provide
the title, dates and web address of each conference, to enable readers to find out more information.
Inclusion does not imply endorsement by the journal. If you know of a relevant conference, please
contact the Managing Editor.
Copyright  2005 John Wiley & Sons, Ltd. Comp Funct Genom 2005; 6: 256-258.

				

